# Plasmid DNA Complexes in Powder Form Studied by Spectroscopic and Diffraction Methods

**DOI:** 10.3390/ma17143530

**Published:** 2024-07-17

**Authors:** Aleksandra Radko, Sebastian Lalik, Natalia Górska, Aleksandra Deptuch, Jolanta Świergiel, Monika Marzec

**Affiliations:** 1 Institute of Physics, Jagiellonian University, Łojasiewicza 11, 30-348 Kraków, Polandsebastian.lalik@uj.edu.pl (S.L.); 2 Faculty of Chemistry, Jagiellonian University, Gronostajowa 2, 30-387 Kraków, Poland; natalia.gorska@uj.edu.pl; 3 Institute of Nuclear Physics Polish Academy of Sciences, Radzikowskiego 152, 31-342 Kraków, Poland; aleksandra.deptuch@ifj.edu.pl; 4 Institute of Molecular Physics Polish Academy of Sciences, Smoluchowskiego 17, 60-179 Poznań, Poland; swiergiel@ifmpan.poznan.pl

**Keywords:** plasmid DNA, DNA complex, dielectric spectroscopy, X-ray diffraction, infrared spectroscopy

## Abstract

Currently, new functional materials are being created with a strong emphasis on their ecological aspect. Materials and devices based on DNA biopolymers, being environmentally friendly, are therefore very interesting from the point of view of applications. In this paper, we present the results of research on complexes in the powder form based on plasmid DNA (pDNA) and three surfactants with aliphatic chains containing 16 carbon atoms (cetyltrimethylammonium chloride, benzyldimethylhexadecylammonium chloride and hexadecylpyridinium chloride). The X-ray diffraction results indicate a local hexagonal packing of DNA helices in plasmid DNA complexes, resembling the packing for corresponding complexes based on linear DNA. Based on the Fourier-transform infrared spectroscopy results, the DNA conformation in all three complexes was determined as predominantly of A-type. The two relaxation processes revealed by dielectric spectroscopy for all the studied complexes are connected with two different contributions to total conductivity (crystallite part and grain boundaries). The crystallite part (grain interior) was interpreted as an oscillation of the polar surfactant head groups and is dependent on the conformation of the surfactant chain. The influence of the DNA type on the properties of the complexes is discussed, taking into account our previous results for complexes based on linear DNA. We showed that the type of DNA has an impact on the properties of the complexes, which has not been demonstrated so far. It was also found that the layer of pDNA–surfactant complexes can be used as a layer with variable specific electric conductivity by selecting the frequency, which is interesting from an application point of view.

## 1. Introduction

The research on deoxyribonucleic acid (DNA) was inextricably linked to and motivated mainly by genetics, while the discovery of other, potentially useful properties of DNA began only in the 21st century. From a chemical point of view, deoxyribonucleic acid belongs to the group of macromolecular organic compounds, the group of nucleic acids. DNA is a linear, unbranched biopolymer, and its monomer consists of 2-deoxyribose (C_5_H_10_O_4_), which connects to the phosphate residue PO_4_ through the 5′ carbon atom [1]. On the other hand, the C1 carbon atom forms an N-glycosidic bond with one of the nitrogenous bases (adenine A, guanine G, thymine T, cytosine C) [2,3]. Linear double-stranded DNA (dsDNA) is the most studied form, but there are also other linear forms: single-stranded (ssDNA), triple-stranded and four-stranded. The double-stranded DNA can take various forms depending on environment (e.g., the humidity, pH, chemical environment or salt content). The most common are two right-handed forms (A-DNA and B-DNA) and one left-handed form (Z-DNA). The main form is B-DNA; the A-DNA form is formed at reduced humidity from the B-DNA form, while the Z-DNA form occurs in an environment with a high salt content. Additionally, DNA can also exist in the form of small circular molecules called plasmids. Plasmid molecules are usually smaller than chromosomal DNA molecules and consist of 300 bp to 2400 kbp (human chromosomal DNA has a length of approx. 4700 kbp—approx. 250 Mbp) [4]. Plasmid DNA can occur in three forms: circular, linear and supercoiled [5].

It should be also noted here that the chemical bonds that make up the individual strands of DNA and the double helix as a whole are weak (e.g., hydrogen bonds between base pairs), but the molecule as a whole is very stable [6]. The helical form of DNA contains large and small grooves in the outer surface of the molecule. In DNA solutions, there are bound water molecules in these grooves. Some cations (counterions) bind to the phosphate backbone with a weak covalent bond. On the other hand, other cations are loosely bound and can penetrate into the grooves of the DNA helix. The counterions bind close to the negative charge centres of the DNA, and a double layer is formed. Ions attracted to the charged DNA backbone form a shell that shields some of the negative charge of the DNA. The dominant charges on the DNA molecule come from the oxygen atoms that are part of the phosphate groups located on the outer surfaces of the double helix. In solutions, the shell around the DNA double helix is usually formed by Na^+^ or Mg^2+^ counterions, which are attracted to the backbone with negatively charged phosphate groups. These counterions have some mobility and oscillate around the phosphate charge centres in an external alternating electric field (measuring field). However, when a cationic surfactant is used, the counterions are surfactant cations, for example, for the surfactant cetyltrimethylammonium chloride (CTMA), it is CTMA^+^.

It is commonly believed that a double-stranded DNA molecule has a permanent dipole moment [6]. The double-stranded DNA macromolecule is negatively charged along the entire length of the double helix due to the presence of phosphate residues. The dipole moment of one strand is opposite to the dipole moment of the other strand. Thus, the net dipole moment of double-stranded DNA should be zero. When DNA is dissolved in a cationic solution, a part of the negative charges is neutralized by the cations. Therefore, the addition of cationic surfactants to the phosphate backbone of the DNA double helix causes symmetry breaking, and a dipole moment is induced. Moreover, the deformation of the DNA molecule by the external alternating electric field applied may cause a non-zero net dipole moment of double-stranded DNA without cationic surfactants.

The very well-known properties of the DNA biomacromolecule cause great interest in this material in various fields of science [7,8,9,10,11]. In recent decades, many applications have been found for it outside of biological sciences, most often in the form of DNA–cationic surfactant complex. Such complexes are characterized by a very high application potential with the possibility of implementation in various fields, from medicine through photonics to organic electronics. Most commonly, such compounds are used in life science to act as drug delivery systems [12]. Outside life science, DNA complexes are currently being used to create new functional materials for applications in, for example, organic thin-film transistors and biosensors [13,14,15,16,17], as well as being a basic building block in the field of supramolecular chemistry for conducting molecular calculations [18,19].

The study of the properties of DNA complexes is therefore very interesting from the point of view of their applications, also considering the ecological aspect of the new functional materials and devices based on them. It is known that complexation with cationic surfactants improves the optical properties of DNA [11]. The question therefore arises whether such a cationic surfactant will change/improve the dielectric properties of the material. There are also many articles that do not specify a DNA type. Our idea is to check whether the type of DNA influences the properties of the complexes. Hence, we synthesized complexes based on linear and plasmid DNA types with three selected surfactants. Moreover, we conducted this research on samples in the form of powder, not solutions. This type of research is not common and that is why we decided to conduct it. The properties of DNA–surfactant complexes in the form of powders based on linear DNA have already been studied by us [20]. In this paper, we describe the properties of DNA–surfactant complexes based on plasmid DNA, compare them with the properties of complexes based on linear DNA and show that the type of DNA has an impact on the properties of the complexes, which has not been demonstrated so far. We investigated the influence of DNA type and surfactant type on the permittivity, specific electric conductivity, relaxation processes and average distances between the aliphatic surfactant chains in the resulting complexes. The research was carried out by means of Fourier-transform middle-infrared (FT-MIR) and frequency-domain dielectric spectroscopy (FDDS) as well as X-ray diffraction (XRD). FT-MIR spectroscopy was used to check whether complexes were formed and what DNA conformation exists in these complexes, while XRD studies were used to find a local packing of DNA helices in the complexes synthesized. In turn, the FDDS method is widely used because it is very sensitive, and the result is obtained quickly. In this method, the sample’s reaction to an applied weak alternating electric field (the so-called measuring field) is recorded. As mentioned above, DNA–surfactant complexes exhibit a dipole moment, and the interaction between the measuring field and the dipole moment can be recorded in FDDS, so this method is useful. In our research, it was used to study the electric properties of the synthesized complexes. The electric properties of the complexes are important because, as previously mentioned, the possibilities of their use in organic thin-film transistors or biosensors are already being investigated.

## 2. Materials and Methods

### 2.1. Materials

Plasmid DNA, phMGFP (Promega, Madison, WI, USA), was isolated from transformed Escherichia coli bacteria with a NucleoBond 10 000 EF DNA extraction kit (Macherey-Nagel, Düren, Germany) as described previously [21]. Cationic surfactants: cetyltrimethylammonium chloride (CTMA, CH_3_(CH_2_)_15_N(Cl)(CH_3_)_3_), hexadecylpyridinium chloride (HDP, C_21_H_38_ClN·H_2_O) and benzyldimethylhexadecyl-ammonium chloride (BAC, CH_3_(CH_2_)_15_N(Cl)(CH_3_)_2_CH_2_C_6_H_5_) as well as Tris base (tris(hydroxymethyl) aminomethane) and hydrochloric acid (HCl) were used as received from Sigma-Aldrich (St. Louis, MO, USA).

The applied cationic surfactants BAC, CTMA and HDP have in their molecular structure an aliphatic chain containing 16 –CH_2_– groups (–(CH_2_)_15_CH_3_); the difference is only in the end of this chain. The BAC surfactant has the highest molecular weight, while the CTMA surfactant has the lowest. For each surfactant, the positive charge is deposited on the nitrogen atom, which, in each case, has a completely different chemical surrounding. It is through this nitrogen atom that the surfactant interacts with the negatively charged double helix of pDNA (or rather, the unsaturated oxygen atoms of the phosphate residues), resulting in a charged double layer and the formation of the complex. The pDNA–surfactant complexes were synthesized according to the procedure described by us in [20].

### 2.2. Methods

Fourier-transform middle-infrared absorption measurements (FT-MIR) were performed at room temperature in transmission mode using a Bruker VERTEX 70v vacuum spectrometer (Billerica, MA, USA). The spectra were obtained in the range of 400–4000 cm^−1^ with a spectral resolution of 2 cm^−1^ and 32 scans per spectrum. Bulk samples of the pure components (dried pDNA, BAC, CTMA and HDP surfactant) as well as the pDNA–surfactant complexes were mixed with KBr, compressed into pellets and measured at room temperature.

X-ray diffraction (XRD) measurements for polycrystalline pDNA complexes (in borosilicate glass capillaries with a 0.5 mm outer diameter) were carried out in the *2θ* = 2–40° range with an Empyrean 2 (PANalytical, Malvern, UK) diffractometer (CuKα radiation, parabolic mirror in the incident beam). The diffraction pattern of an empty capillary was used for background subtraction. The results were analysed using WinPLOTR [22] and Origin.

Frequency domain dielectric spectroscopy (FDDS) measurements were performed by using the Turnkey Impedance Spectrometer Concept 81 (Novocontrol Technologies GmbH & Co. KG, Montabaur, Germany) at room temperature (23.0 ± 0.5 °C) in the frequency range of 0.5 Hz–3.0 MHz, at a measuring voltage of 0.1 V. All pellet samples were placed in a flat, rounded electrode capacitor (diameter of 9.60 mm). The thickness of each sample was determined by a micrometric screw (see Table 1).

## 3. Results and Discussion

**Figure 1:**
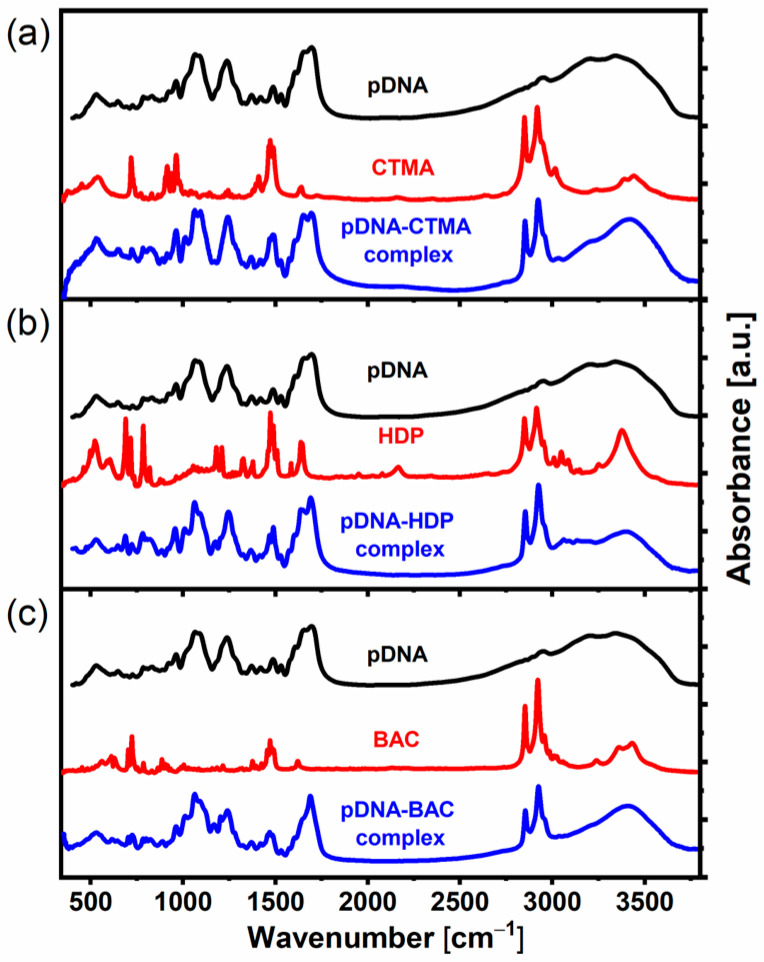
FT-MIR spectra of pure components (pDNA, CTMA, HDP and BAC) and obtained complexes: pDNA–CTMA (**a**), pDNA–HDP (**b**) and pDNA–BAC (**c**).

The FT-MIR spectra of the pure components: plasmid DNA, CTMA, HDP and BAC surfactants as well as the synthesized plasmid DNA–surfactant complexes are presented in Figure 1. In the spectrum of pure plasmid DNA, the bands at 832, 962, 1290 and 1418 cm^−1^ characteristic for the B-conformation of DNA are present. In addition to them, the bands that are spectroscopic markers for the A-DNA form are observed at 801, 860, 1176 and 1238 cm^−1^. There is also one band visible at 1208 cm^−1^, which, in turn, can be associated with the Z-DNA form. Thus, the pure plasmid DNA substrate used in our study was most probably a mixture of the three conformations, with the B and A forms being dominant. The molecular structure of pDNA differs from that observed for linear DNA substrate used in preparation of the analogical DNA–surfactant complexes presented in our previous work, in which the B-DNA form was predominant [20]. A comparison of the IR spectra of bulk plasmid and linear DNA along with a description of their bands is presented in the Appendix A.

In the case of the DNA–surfactant complex formation, the FT-MIR spectra show characteristic changes in the positions and/or intensities of some selected bands of molecular groups of both DNA and surfactant sensitive to moderate structural and environmental modifications. Taking into consideration the DNA and cationic surfactant molecules, the predicted complex formation is based on electrostatic interactions between nitrogen—containing a polar surfactant head group and the negatively charged phosphate group of the DNA backbone. As we expect, a shift in the band connected to the *ν*_as_(PO_2_^−^) mode toward larger wavenumbers (above 1240 cm^−1^) within the spectral range characteristic for A-type DNA conformation was observed in case of all three obtained complexes, which proves that this group is affected during complexation. Also, the band associated with the *ν*_as_(PO_2_^−^) mode for the Z-DNA form and observed in pure pDNA at 1208 cm^−1^ shifted toward smaller wavenumbers by 7 cm^−1^ in case of pDNA–HDP and pDNA–BAC complexes. The bands connected to *ν*_s_(CH_2_) and *ν*_as_(CH_2_) stretching modes of hydrocarbon chains of all three surfactants (CTMA, HDP and BAC) shifted toward larger wavenumbers on the complex formation, indicating their slightly larger degree of disorder comparing to pure surfactants. This is consistent with our previous study [20]. The most significant changes in the IR band positions of the pDNA–surfactant complexes in comparison to pure components indicating molecular modifications occurring in the structure on complexation are summarized in Table 2.

Summing up, the FT-MIR spectroscopic results are very consistent in the case of all the three obtained complexes, which indicates that the mechanism of their formation is the same and based on electrostatic interactions. The positions of the bands connected to the asymmetric phosphate (1243–1246 cm^−1^) and phosphodiester (intense band at 1062 cm^−1^) modes indicate that, upon complexation, most probably, the A-DNA conformation of double helix was predominant.

**Figure 2:**
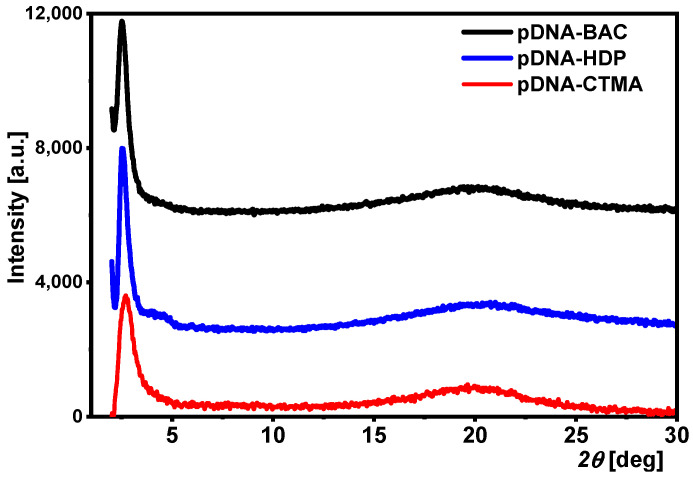
XRD patterns for pDNA-based complexes with three different surfactants.

The diffraction patterns for the pDNA complexes (Figure 2) show a sharp peak at low angles *2θ* ≈ 2.5–2.7° and a wide maximum at about *2θ* ≈ 20°. The latter corresponds to an average distance of 0.44–0.46 nm between the aliphatic chains of surfactants. The position of the peak at low angles corresponds to the distance 3.2–3.5 nm (Table 3), which can be interpreted as the distance *d*_02_ *= d*_11_ in the two-dimensional centred rectangular lattice (see Figure 5 in [20]) with the unit cell parameters *a* = *b*/√3 and *b* = 2*d*_02_. The condition *a* = *b*/√3, resulting in the overlapping of the (02) and (11) diffraction peaks, indicates the hexagonal packing of the DNA helices, with the distance between the nearest neighbours equal to *a* = 3.7–4.0 nm. The differences in the size of the unit cell arise from the different lengths of the surfactants’ molecules.

The diffraction patterns recorded for the plasmid DNA complexes are similar to those obtained for the complexes synthesized based on linear DNA [20]. The structures formed by the circular and supercoiled forms of DNA are too large (the estimated hydrodynamic diameter of the circular plasmid form, approx. 4500 bp, is 320 nm [26]) to be observed in the diffraction patterns obtained by the standard XRD method, which allows the study of structures with sizes up to approx. 18 nm (distance corresponding to the angle *2θ* ≈ 0.5° for CuKα radiation).

**Figure 3:**
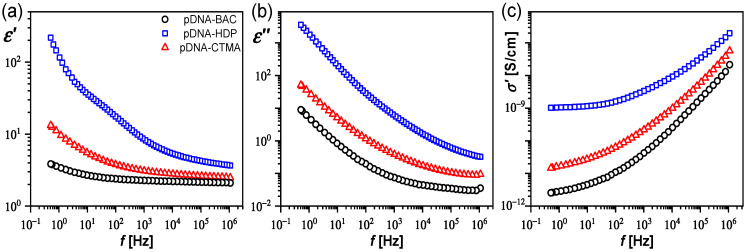
The dielectric dispersion (**a**) and absorption (**b**) as well as real part of specific electric conductivity (**c**) for pDNA complexes. The legend in (**a**) is the same for all graphs.

As it was mentioned above, the dielectric properties of the pure surfactant and its complexes with linear DNA were described in our previous paper [20]. Here, we present and discuss the dielectric spectra of plasmid DNA complexes with three surfactants (Figure 3a,b). The smallest dielectric dispersion *ε′* and absorption *ε″* as well as the smallest real part of specific electric conductivity *σ′* in the entire studied frequency range were recorded for the pDNA–BAC complex, while the highest belonged to the pDNA–HDP complex. Similarly, the smallest dielectric dispersion *ε′* was registered for the complex based on the linear DNA with the BAC surfactant (dsDNA–BAC) [20]. Thus, the type of surfactant, and/or its conformation, affects the dielectric dispersion *ε′* in complexes, similar to DNA solutions, where *ε′* depends on the type of cations and concentration (the higher the concentration of cations, the more phosphate groups are neutralized and the dielectric dispersion decreases) [6]. In turn, the dielectric absorption *ε″* increases as the frequency decreases for all the pDNA-based complexes, and it is the highest for the pDNA–HDP complex and about one order of magnitude greater at the low frequency limit than for the pDNA–CTMA complex (Figure 3b). This behaviour is fundamentally different from that of the complexes based on linear DNA in which the dielectric absorption was constant over the entire frequency range and equal for both dsDNA–CTMA and dsDNA–HDP complexes [20]. Taking into account that the dielectric absorption for pDNA–HDP is about 3000 higher than that for dsDNA–HDP at the lowest frequency, it can be concluded that the type of DNA in the complex has a large impact on the dielectric properties.

On the other hand, the real part of the specific electric conductivity *σ′* increases with increasing frequency for all the studied complexes, and, for selected frequencies (0.5 and 50.0 Hz), it is about three orders of magnitude lower for pDNA–BAC than for pDNA–HDP (Figure 3c, Table 4). Thus, the layer of pDNA–surfactant complexes can be used as a layer (or dielectric layer in capacitor) with a specific electric conductivity (or capacitance), which can be changed by selecting the appropriate surfactant. In addition, since the specific electric conductivity of pDNA complexes varies strongly with frequency (about three orders of magnitude), they can be used as a layer with a variable conductivity *σ′* by frequency selection (see Table 4).

**Figure 4:**
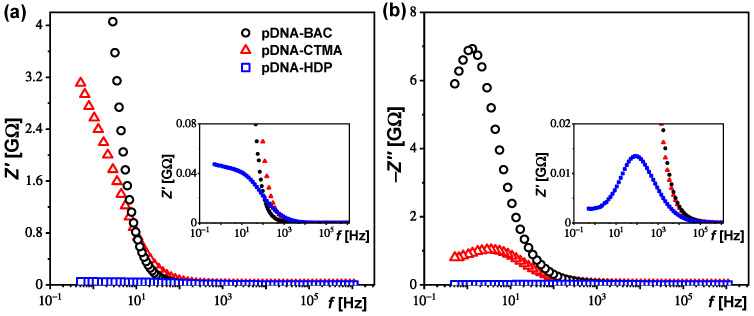
Real (**a**) and imaginary (**b**) part of the impedance for all complexes studied; legend in (**a**) is valid for (**b**).

**Figure 5:**
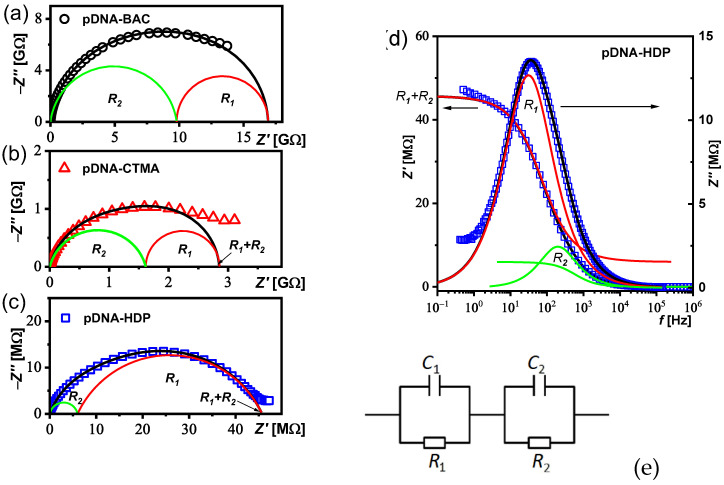
Nyquist plot for complexes studied: pDNA–BAC (**a**), pDNA–CTMA (**b**) and pDNA–HDP (**c**) as well as an impedance spectrum with fitting result for pDNA–HDP complex (**d**). Black line represents the best fit of Equation (1) to the experimental data (open points); the red line represents fitted data for the grain boundaries (*R*_1_), while the green line represents the grain interior (crystallite part of the sample—*R*_2_). Calculated equivalent circuit: two RC parallel connected in series (**e**).

Since for the pDNA–HDP complex a weak relaxation process is visible around 10–200 Hz on the dielectric dispersion (Figure 3a), the dielectric spectra of all complexes studied (*ε′*(*f*), *ε″*(*f*)) were transformed to the impedance representation (*Z′*(*f*), *Z″*(*f*)) for its analysis. Figure 4 presents the real and imaginary parts of the impedance, while Nyquist plots are presented in Figure 5a–c. The relaxation process at the low frequency range is clearly visible for all the studied complexes.

The Cole–Cole model [27] was fitted to the experimental data. The measured complex impedance Z*(*ω*) can be described by an equivalent circuit consisting of two parallel RC circuits connected in series:

(1) $$Z^{*}\left(\omega \right)=\frac{R_{1}}{1+{\left(i\omega R_{1}C_{1}\right)}^{1-\alpha_{1}}}+\frac{R_{2}}{1+{\left(i\omega R_{2}C_{2}\right)}^{1-\alpha_{2}}}$$

where *R*_i_ is the sample direct current resistance of the *i*-th contribution; *C*_i_ is the electric capacity of the *i*-th contribution and α_i_ (0 < α ≤ 1) is a parameter of the relaxation time distribution, an empiric exponent of the *i*-th contribution related to the deviation from the Debye model. ω = 2π*f* is angular frequency, *f*—frequency of measuring AC electric field. The black line in Figure 5a–d represents the best fit of Equation (1) to the experimental data with the low- (red line—*R*_1_) and high-frequency (green line—*R*_2_) contributions. The fitting parameters obtained for all complexes studied are gathered in Table 5. As can be seen, the sum *R*_1_ *+ R*_2_ was three orders of magnitude greater for pDNA–BAC (or pDNA–CTMA) than for the pDNA–HDP complex. A response consisting of two semicircles is often observed in polycrystalline samples, and it is related to two different contributions to total conductivity: conductivity of the crystallite part (high-frequency contribution—*R*_2_) and the conductivity of the grain boundaries (low-frequency contribution—*R*_1_) [28]. In our case, the samples are in the form of powder, so both contributions are possible. Based on the analysis presented in Figure 5, it can be concluded that the grain boundaries’ contribution (*R*_1_) exists for all the studied complexes based on plasmid DNA. 

As we have shown in our previous paper [20], the highest specific electric conductivity *σ′* in the whole frequency range occurred for the BAC surfactant, while, for CTMA and HDP, it was lower and comparable (which probably depends on the position of the nitrogen atom and/or the presence of an additional –CH_2_– group between the nitrogen atom and the benzene ring). The specific electric conductivity for the pDNA–CTMA and pDNA–HDP complexes increased relative to that of the pure surfactants, while for pDNA–BAC a slight decrease relative to pure BAC was visible. In turn, the *σ’* was the lowest for complexes with the BAC surfactant (linear dsDNA–BAC and plasmid pDNA–BAC), which may result from the conformation of the BAC chains when interacting with the negatively charged DNA double helix. This double helix has a negative charge coming from phosphoric acid (V) residues, where each phosphate residue has an unsaturated oxygen atom. Thus, negative charges on phosphate residues are the cause of electrostatic interaction (even the formation of weak covalent chemical bonds) between the DNA backbone and the cationic surfactant.

To find the origin of the second registered relaxation process (conductivity of the crystallite part, high-frequency contribution—*R*_2_) in the pDNA–surfactant complexes studied, let us consider DNA solutions for a moment. In DNA solutions containing cations (counterions) such as Na^+^ or Mg^2+^, some of these cations are bound to the phosphate backbone by a weak chemical bond, while other cations are more loosely bound and can move around the helix. Cations attracted to the phosphate backbone form a cationic (counterionic) shell that shields part of the negative charge of the double helix. As a result, a double charge layer (the Stern layer) is formed [6]. The attracted cations show some mobility and oscillate around the phosphate charge centres in alternating electric fields (it is measuring field during experiments). These cationic oscillations represent a relaxation process that is visible at the low frequency range (1–100 Hz). In turn, the relaxation process associated with the movement of counterions bound to individual phosphate groups appears in the range of several MHz. Other relaxation processes occur at frequencies above hundreds of GHz, e.g., propeller twist relaxation, which occurs when two adjacent nitrogen bases in a pair twist in opposite directions, or breather relaxation, where two nitrogen bases oscillate in opposite to each other, which causes the hydrogen bonds to be alternately shortened and lengthened [6].

Although our study did not deal with DNA solutions but with pDNA–surfactant complexes in powder form, we believe that the charge double layer was formed in the same way and that the oscillating polar surfactant head groups were responsible for the relaxation process seen in at the higher frequency range. It is possible that during this relaxation process the aliphatic chain also oscillates/fluctuates, but we did not record any relaxation processes for pure surfactants [20]. The influence of the molecular weight of the surfactant on the relaxation time *τ*_2_ of this process should also be considered (for the surfactant with the highest molecular weight (BAC), the relaxation time *τ*_2_ should be the longest and the relaxation frequency the smallest, which we actually observed, see Table 5). Therefore, the shortest relaxation time should be for the pDNA–CTMA complex. However, the shortest relaxation time *τ*_2_ was found for the pDNA–HDP complex. Thus, assuming that the relaxation process revealed at the high frequency range is of the same origin for all the studied pDNA-based complexes, the molecular weight of the cationic surfactant is not a dominant factor influencing the relaxation time of this process. It seems, however, that the conformation of the surfactant chain, which enforces the degree of packing, has a much greater impact. Finally, it cannot be ruled out that the relaxation time may also be affected by the purity of the surfactants and the residue of unwashed residual chloride ions (stabilizing pure surfactants).

## 4. Conclusions

Complexes based on plasmid DNA and three surfactants with aliphatic chains containing 16 carbon atoms (BAC, CTMA and HDP) in the powder form have been studied by Fourier-transform middle-infrared spectroscopy, X-ray diffraction and frequency-domain dielectric spectroscopy methods. Based on the results obtained, the following conclusions can be drawn:

- The DNA conformation in all three plasmid DNA complexes was found to be predominantly of the A type, although the plasmid DNA used to form the complexes was most likely a mixture of three conformations (A-DNA, B-DNA and Z-DNA) with B and A as the predominant forms. After complexation, the A-DNA double-helix form predominated in plasmid-based pDNA complexes, similarly to complexes based on linear dsDNA (although linear dsDNA with a predominant B-DNA form was used [20]).
- The pDNA helices in all studied complexes were locally packed into a hexagonal lattice, similarly to what was found in complexes of linear DNA with these surfactants (BAC, CTMA and HDP) [20]. The fact that the plasmid DNA was in a form of a closed loop did not prevent the hexagonal packing because the length of the used DNA (320 nm) was almost two orders of magnitude larger than the distances between neighbouring helices (3.7–4.0 nm).
- In contrast to linear DNA complexes studied by us earlier [20], two relaxation processes were revealed for plasmid DNA complexes (contributions to total conductivity coming from conductivity of the crystallites (grain interior) and from conductivity of the grain boundaries). The relaxation process in the crystallites revealed for all the studied complexes was interpreted as an oscillation of the polar surfactant head groups and seemed to be independent of the molecular weight of the cationic surfactant but dependent on the conformation of the surfactant chain.
- The dielectric absorption changed in a completely different way for complexes based on plasmid pDNA and linear dsDNA. It increased with decreasing frequency for all the pDNA-based complexes, whereas it was constant over the entire frequency range for all the dsDNA-based complexes [20]. Moreover, the dielectric absorption for pDNA–HDP was about 3000 higher than that for for dsDNA–HDP.
- The specific electric conductivity depended on the surfactant forming the complexes based on plasmid pDNA, as it was found earlier for linear dsDNA. In addition, it seems that the conformation of the BAC chains caused *σ’* to be the lowest for complexes with the BAC surfactant for both linear dsDNA and plasmid pDNA.

Summarizing, we showed that the type of the DNA (plasmid or linear) had a great influence on the dielectric properties of the complexes with cationic surfactants. The results of our study show that the specific electric conductivity of the layer of pDNA–surfactant complex can be changed by selecting the frequency, which is interesting from an application point of view. Considering that more and more attention is paid to recycling and biodegradability when designing new functional materials and that large-scale research into conductive biodegradable materials is currently underway, a complex based on plasmid DNA with a cationic surfactant seems to be a promising candidate. However, this issue requires further research, such as studying the influence of temperature and environment on the properties of these complexes.

## Figures and Tables

**Table 1:** The thickness of the studied pDNA–surfactant complex.

Sample	Thickness [mm]
pDNA–BAC	0.30(1)
pDNA–HDP	0.35(1)
pDNA–CTMA	0.35(1)

**Table 2:** Comparison of the selected FT-MIR bands’ positions (in cm^−1^) for pure pDNA, CTMA, HDP, BAC and the pDNA–surfactant complexes with their tentative assignment based on the literature [23,24,25].

pDNA	CTMA	HDP	BAC	pDNA–CTMA	pDNA–HDP	pDNA–BAC	Assignment
	719 shp	717 shp	723 shp	724 br	722 br	725 br	*ρ*(CH_2_)_al,n_
801 ^A^				800 ^A^	800 ^A^	801 ^A^	Sugar—phosphate backbone
832 ^B^				822 ^B^	825 ^B^	823 ^B^	Sugar—phosphate backbone
860 ^A^				–	–	–	Sugar—phosphate backbone
962 ^B^				960	957	960	*ν*(C-C) of DNA backbone
~1015 sh				1011 shp	1009 sh	1010 shp	Furanose ring
1063				1062	1062	1062	*ν*(C-O) phosphodiester linkage
1088				1094	1092	1091	*ν*_s_(PO_2_^−^)
1176 ^A^				1168 ^A^	1172 ^A^	1166 ^A^	Sugar base
1208 ^Z^				–	1201 ^Z^	1201 ^Z^	*ν*_as_(PO_2_^−^)
1238 ^A^				1243 ^A^	1246 ^A^	1244 ^A^	*ν*_as_(PO_2_^−^)
~1284 ^B^				1274 ^A^	1278 ^A^	1278 ^A^	Sugar base (T)
1418 ^B^				1418 ^B^	1415 ^B or Z^	1415 ^B or Z^	Sugar—phosphate backbone
	1471	1472	1471	1470	1468	1467	*ν*(C=C)_ar_
		1508	1490	1485	1504	1484	*ν*(C=C)_ar_ or base ring
1531				1527	1528	1531	Deoxycytidine
1696				1690	1690	1689	Base double bond stretch
	2849	2849	2852	2854	2853	2854	*ν*_s_(CH_2_)_al_
	2917	2914	2921	2925	2925	2924	*ν*_as_(CH_2_)_al_

^A, B or Z^ Positions of the IR bands characteristic for A, B or Z conformation of DNA. Mode description: *ν*—stretching; *ρ*—rocking; s—symmetric; as—asymmetric; ar—aromatic; al—aliphatic; n—long chain; sh—shoulder; shp—sharp; br—broad; T—thymine.

**Table 3:** The maxima position for pDNA complexes and characteristic distances calculated on the basis of the Bragg equation.

	Low-Angle Peak		Peak at ca. 20°	
	*2θ* [deg]	*d* [nm]	*2θ* [deg]	*d* [nm]
pDNA–CTMA	2.77(1)	3.19(2)	19.69(3)	0.46(1)
pDNA–BAC	2.52(1)	3.50(2)	19.88(2)	0.45(2)
pDNA–HDP	2.60(1)	3.39(2)	20.54(2)	0.44(2)

**Table 4:** The dielectric dispersion and specific electric conductivity at two frequencies for the complexes studied.

	*f* = 0.5 Hz		*f* = 50.0 Hz	
	*ε′*	*σ′* [pS/cm]	*ε′*	*σ′* [pS/cm]
pDNA–BAC	3.85	2.55	2.44	7.68
pDNA–CTMA	13.25	14.57	4.11	47.79
pDNA–HDP	217.72	1019.8	21.87	1388.70

**Table 5:** Fitting parameters obtained from the impedance spectra analysis.

	pDNA–BAC	pDNA–CTMA	pDNA–HDP
*R*_1_ [GΩ]	7.10 ± 0.07	1.24 ± 0.02	(39.77 ± 0.09) · 10^−3^
*τ*_1_ [s]	0.2204 ± 0.0049	0.0761 ± 0.0015	(2.2321 ± 0.0004) · 10^−3^
*C*_1_ [pF]	31.04 ± 0.25	61.35 ± 0.65	303.83 ± 1.18
*α*_1_	0	0	0.277 ± 0.002
*R*_2_ [GΩ]	9.81 ± 0.04	1.62 ± 0.01	(5.93 ± 0.05) · 10^−3^
*τ*_2_ [s]	0.0769 ± 0.0021	0.0181 ± 0.0005	(2.6998 ± 0.0002) · 10^−4^
*C*_2_ [pF]	9.65 ± 0.05	20.74 ± 0.15	124.86 ± 3.13
*α*_2_	0.081 ± 0.001	0.154 ± 0.003	0.124 ± 0.004

**Figure A1:**
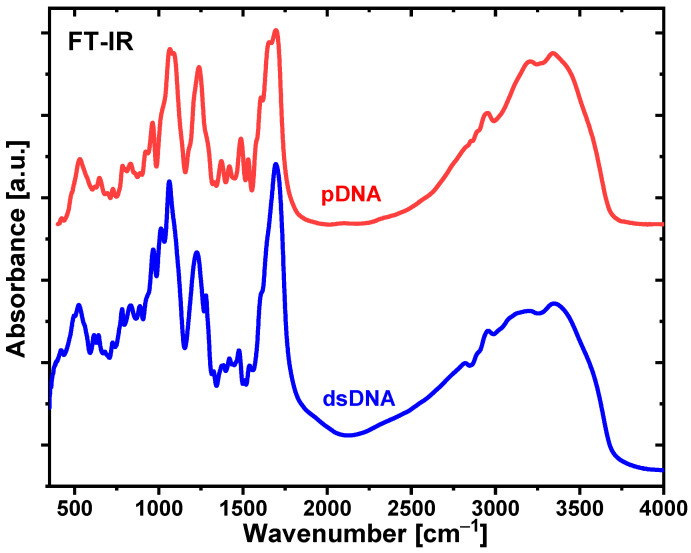
Comparison of FT-MIR spectra of bulk plasmid DNA (pDNA) and linear DNA (dsDNA). Results for linear DNA are presented in [20].

## Data Availability

Data are contained within the article and the Appendix A.

## Appendix A

FT-MIR Spectroscopy

Both FT-MIR spectra presented here were obtained at room temperature with a spectral resolution of 2 cm^−1^ and 32 scans per spectrum in transmission mode using a Bruker VERTEX 70v vacuum spectrometer. Bulk samples of the dried plasmid DNA and linear DNA were mixed with KBr, compressed into pellets and measured. Additionally, parallel to these measurements, the FT-ATR reference spectra were collected for comparison to ensure that the compression did not damage the DNA material.
